# The docking protein p130Cas regulates cell sensitivity to proteasome inhibition

**DOI:** 10.1186/1741-7007-9-73

**Published:** 2011-10-28

**Authors:** Ming Zhao, Kristiina Vuori

**Affiliations:** 1 Cancer Center, Sanford-Burnham Medical Research Institute, 10901 N. Torrey Pines Road, La Jolla, CA 92037, USA

## Abstract

**Background:**

The focal adhesion protein p130Cas (Cas) activates multiple intracellular signaling pathways upon integrin or growth factor receptor ligation. Full-length Cas frequently promotes cell survival and migration, while its C-terminal fragment (Cas-CT) produced upon intracellular proteolysis is known to induce apoptosis in some circumstances. Here, we have studied the putative role of Cas in regulating cell survival and death pathways upon proteasome inhibition.

**Results:**

We found that Cas-/- mouse embryonic fibroblasts (MEFs), as well as empty vector-transfected Cas-/- MEFs (Cas-/- (EV)) are significantly resistant to cell death induced by proteasome inhibitors, such as MG132 and Bortezomib. As expected, wild-type MEFs (WT) and Cas-/- MEFs reconstituted with full-length Cas (Cas-FL) were sensitive to MG132- and Bortezomib-induced apoptosis that involved activation of a caspase-cascade, including Caspase-8. Cas-CT generation was not required for MG132-induced cell death, since expression of cleavage-resistant Cas mutants effectively increased sensitivity of Cas-/- MEFs to MG132. At the present time, the domains in Cas and the downstream pathways that are required for mediating cell death induced by proteasome inhibitors remain unknown. Interestingly, however, MG132 or Bortezomib treatment resulted in activation of autophagy in cells that lacked Cas, but not in cells that expressed Cas. Furthermore, autophagy was found to play a protective role in Cas-deficient cells, as inhibition of autophagy either by chemical or genetic means enhanced MG132-induced apoptosis in Cas-/- (EV) cells, but not in Cas-FL cells. Lack of Cas also contributed to resistance to the DNA-damaging agent Doxorubicin, which coincided with Doxorubicin-induced autophagy in Cas-/- (EV) cells. Thus, Cas may have a regulatory role in cell death signaling in response to multiple different stimuli. The mechanisms by which Cas inhibits induction of autophagy and affects cell death pathways are currently being investigated.

**Conclusion:**

Our study demonstrates that Cas is required for apoptosis that is induced by proteasome inhibition, and potentially by other death stimuli. We additionally show that Cas may promote such apoptosis, at least partially, by inhibiting autophagy. This is the first demonstration of Cas being involved in the regulation of autophagy, adding to the previous findings by others linking focal adhesion components to the process of autophagy.

## Background

The ubiquitin-dependent proteasome system maintains normal cellular function by eliminating aberrant proteins in response to extracellular stimuli. Proteasome inhibition has also emerged as part of adjuvant therapy to increase sensitivity of cancer cells to chemotherapeutic agents. Recently, the proteasome inhibitor Bortezomib (Velcade™) was approved by the FDA for treatment of relapsed multiple myeloma and mantle cell lymphoma. Mechanisms underlying proteasome inhibition-mediated cell death are not well understood.

Focal adhesion protein p130Cas (Cas) is a multi-domain docking protein, with an N-terminal SH3 domain, a central "substrate domain" consisting of multiple SH2-domain binding motifs (Tyr-x-x-Pro), and a "Src-binding domain" near the C-terminus [1]. Following integrin activation or growth factor stimulation, Cas interacts with several protein partners and regulates cellular events, such as migration, proliferation, and survival [2]. Most notably, the adaptor protein Crk is a primary docking partner and its association with phosphorylated Cas induces Rac-dependent cell migration and promotes tumor cell invasion [3]. In addition to regulating cell motility, Cas can also relay survival signals from the extracellular matrix to the nucleus [2]. It has been reported that Cas confers resistance to anticancer drugs such as Doxorubicin and tamoxifen via activation of signaling pathways mediated by various kinases, including Src, EGFR, Akt and ERK1/2 [4,5]. Ta *et al*. observed that human breast cancer cell lines demonstrate varying sensitivity to Doxorubicin, possibly due to variable expression levels of Cas [5]. In fact, Cas expression is considered a useful prognostic marker for patients with primary or metastatic breast cancer [4]. Signaling via the Cas/Crk complex also promotes cell survival via activation of ERK and Rac pathways [6,7], and it has been reported that uncoupling of Cas and Crk is required for Abl-mediated apoptosis to take place [8].

While Cas frequently appears to signal pro-survival, increasingly literature demonstrates that the C-terminal domain of Cas that is released by proteolysis may play an opposing role. For example, etoposide treatment of HeLa cells elicits apoptosis accompanied by Cas cleavage, yielding a C-terminal 31 kD fragment (Cas-CT). Specifically, Cas exhibits cleavage first at site Asp^416 ^yielding a C-terminal 74 kD product. Subsequent cleavage of this fragment at Asp^748 ^results in the generation of the C-terminal 31 kD fragment during etoposide-induced apoptosis, and replacement of Asp^748 ^with a glutamic acid residue blocks the 31 kD Cas-CT production in this system [9]. Notably, the 31 kD fragment has been found to physically interact with the transcription factor E2A in the nucleus and repress its activity, preventing E-box binding to E2A and thus inhibiting E2A-mediated p21^WAF1/CIP1 ^transcription and promoting apoptosis [10]. Similar observations have been reported on the death-promoting ability of another Cas family member, human enhancer of filamentation (HEF1), which requires generation of a C-terminal 28 kD fragment [11].

Several additional pro-apoptotic stimuli, such as detachment-induced cell death (also known as anoikis), UV irradiation and treatment with anticancer drugs, can induce Cas cleavage as well [12-14]. Both caspase and calpain inhibitors can each partially block Cas cleavage, but cleavage is not completely inhibited when these two inhibitors are combined [9,14], suggesting that additional mechanisms are involved in Cas cleavage. This notion is supported by a study demonstrating that Smad3 interacts with HEF1 and mediates its cleavage upon TGF-β stimulation [15]. In this case, HEF1 degradation was inhibited by the proteasome inhibitor lactacystin but not by the caspase inhibitor Z-VAD.

Here, we used several different cell death assays to show that Cas is crucial for mediating cell death that takes place upon proteasome inhibition. We found that proteasome inhibition induces cell death in cells that express Cas, but not in those that lack Cas expression. Interestingly, inhibition of autophagy in Cas -/- MEFs enhanced proteasome inhibition-induced cell death in these cells. We also demonstrate that accumulated Cas-CT plays only a minor role in MG132-induced cell death. Thus, our studies uncover a novel role for Cas in mediating proteasome inhibition-induced cell death, and suggest that Cas may do so, at least partially, by inhibiting autophagy. Intriguingly, we found that reduced levels of Cas offer protection against cell death induced by the DNA-damaging agent Doxorubicin, too, and this protection again coincides with induction of autophagy. Combined with the findings by Ta *et al*. noting that overexpression of Cas results in chemoresistance [5], our results suggest that Cas may have a universal yet complex regulatory role in cell death signaling in response to multiple different stimuli, perhaps determined by Cas gene/protein dosage.

## Results

### Cas mediates proteasome inhibition-induced cell death

**Figure 1:**
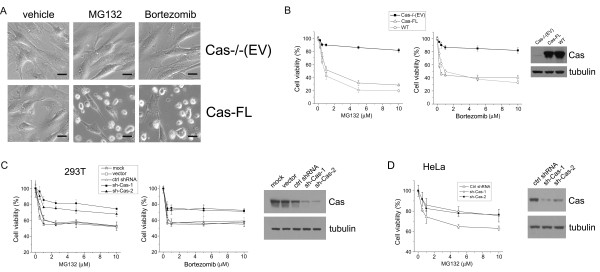
**Cas is required for proteasome inhibition-induced cell death**. **(A) **Cas-/- (EV) and Cas-FL cells cultured in 60 mm dishes were treated with vehicle, or MG132, or Bortezomib overnight, and the images were obtained under phase contrast microscope. Scale bar, 25 μm. **(B) **Cell viability measurement by MTS assay. MEFs grown in 96-well plates were treated with different concentrations of the proteasome inhibitors MG132 or Bortezomib for 16 hours. A total of 20 μl of MTS reagent was added to each well and incubated for another two hours. Absorbance at 490 nm was detected on a plate reader. The data presented depict the MEAN ± SE from three independent experiments. **(C **and **D)**, 293T cells (C) or HeLa (D) cells transiently transfected with Cas shRNAs, control shRNA or empty vector, were treated with MG132 or Bortezomib for 16 hours, then assayed and results are presented as in panel A. shRNA-mediated reduction of Cas expression is shown in immunoblot.

Cas has been shown to regulate cell death pathways in response to various anticancer agents, and we, therefore, decided to examine its potential role in proteasome inhibition-mediated cell death. To determine the role of Cas in proteasome inhibition-induced cell death, Cas -/- MEFs that had been stably transfected with either empty vector (Cas-/- (EV)), or full-length wild-type Cas (Cas-FL), were treated with proteasome inhibitors MG132 or Bortezomib. As expected, MG132 or Bortezomib-treated Cas-FL cells became round and detached under phase-contrast microscope, indicative of proteasome inhibition-induced cell death. Surprisingly, Cas-/- (EV) cells were found to be resistant to proteasome inhibitor-induced cell death (Figure 1A). To further compare the sensitivity to proteasome inhibitors of Cas-/- (EV) and Cas-FL cells, we undertook MTS assay measurements and found that both MG132 and Bortezomib effectively killed wild-type mouse embryonic fibroblasts (WT MEFs) and Cas-FL MEFs in a dose-dependent fashion, while Cas-/- (EV) cells were relatively resistant to these compounds (Figure 1B). To control for differences related to selection of stably transfected clonal cell lines, we also assayed non-transfected primary Cas-/- MEFs and observed effects similar to Cas-/- (EV) cells following treatment with various concentrations of MG132 or Bortezomib (data not shown). Importantly, proteasome activity assay demonstrated that proteasome activity is equally inhibited in Cas-/-(EV) and Cas-FL cells in response to MG132 treatment. As such, differences in drug update or metabolism do not appear to contribute to the cell survival differences observed in the two cell types (data not shown).

To confirm the results observed in MEFs, we used shRNAs to specifically knock-down Cas expression in human 293T and HeLa cells. As shown in Figure 1C, D, two independent Cas shRNAs reduced Cas expression by about 65% in the two cell lines. Concomitant with this, about 50% reduction (demonstrating cell viability percentile change from 60% to 80%, and cell death percentile change from 40% to 20%) was observed in MG132-induced cell death, when Cas shRNA-transfected 293T cells were compared to mock-transfected cells or to cells transfected with empty vector or control shRNA (Figure 1C). Likewise, Bortezomib-induced cell death was reduced by about 45% in Cas shRNA-transfected 293T cells (demonstrating cell viability percentile change from 55% to 75%, and cell death percentile change from 45% to 25%) (Figure 1C). Similarly, knock-down of Cas by shRNA reduced MG132-induced cell death by about 40% (demonstrating cell viability percentile change from 65% to 80%) in HeLa cells (Figure 1D). Thus, these results suggest that proteasome inhibition-induced cell death takes place in Cas-dependent manner in different cell lines.

Our preliminary studies demonstrated that Cas levels may control cellular responsiveness to other apoptotic stimuli, too. While Ta *et al*. have found that overexpression of Cas confers resistance to Doxorubicin in breast cancer cells [5], we found that complete lack of Cas expression also reduces cellular responsiveness to Doxorubicin, although not to the same extent than cellular responsiveness to proteasome inhibitors. Thus, about 37% reduction (demonstrating cell viability percentile change from 45% to 65%, and cell death percentile change from 55% to 35%) was observed in 2.5 μM Doxorubicin-induced cell death, when Cas-/- (EV) cells were compared to Cas-FL cells (Additional file 1). These preliminary results suggest that the role of Cas in drug resistance may be more universal, but also more complicated than previously anticipated, and suggest the intriguing possibility that gene and protein dosage may be emerging as a major mechanism to control distinct biological events in case of Cas (see Discussion).

### MG132-induced cell death is due to apoptosis

**Figure 2:**
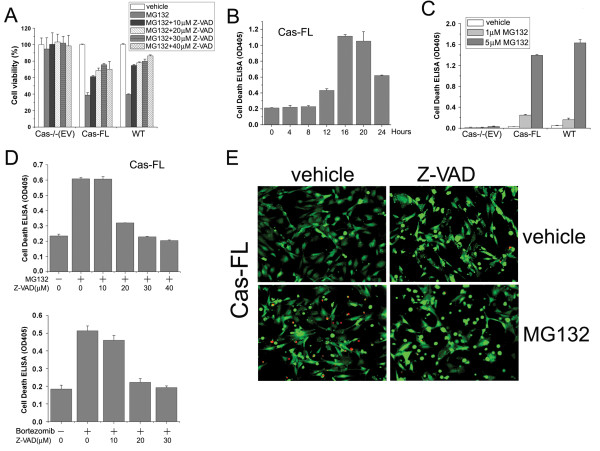
**MG132 induces apoptosis in Cas-expressing cells**. **(A) **MTS assay to determine viability of Cas-/- (EV), Cas-FL and WT MEFs treated with 1 μM MG132 and various concentrations of Z-VAD-FMK for 16 hours. **(B) **Apoptosis assay. Cas-FL MEFs were treated with 1 μM MG132 for different times and lysed. A total of 5 μg of protein from each lysate was used in the cell death ELISA assay (see "Methods"). Results are means ± SE from three independent experiments. **(C) **Cas-/- (EV), Cas-FL and WT MEFs were treated with 1 or 5 μM MG132 for 16 hours, and apoptosis was monitored as in B. **(D) **Cas-FL MEFs were treated for 16 hours with MG132 (upper panel) or Bortezomib (lower panel) concomitant with increasing concentrations of Z-VAD-FMK as indicated, and apoptosis was monitored as above. **(E) **Cell viability assay by live-dead staining. Cas-FL MEFs treated with MG132 ± 30 μM Z-VAD-FMK for 16 hours were stained with calcein (green) and ethidium homodimer (EthD, red), and images were obtained under fluorescence microscope.

Proteasome inhibition reportedly induces cell death by activating cell type-dependent signaling pathways. To determine pathways underlying cell death in MG132-treated MEFs, we treated Cas-FL and WT MEFs with the pan-caspase inhibitor Z-VAD-FMK and observed that the treatment largely prevented MG132-induced cell death in a dose-dependent manner (Figure 2A), suggesting that caspase-mediated apoptosis causes MG132-induced cell death in this system. An ELISA assay measuring nuclear DNA fragmentation as an indication of apoptosis demonstrated that detectable apoptosis takes place within 12 hours in MG132-treated Cas-FL cells, and that the observed apoptosis was at maximal levels 16 hours post-treatment (Figure 2B). Using the same assay, we also observed that both 1 μM and 5 μM MG132 could induce apoptosis in both Cas-FL and WT MEFs, but not in Cas-/- (EV) cells, indicating that MG132-elicited cell apoptosis requires Cas expression (Figure 2C).

We next treated Cas-FL cells with different concentrations of Z-VAD-FMK to examine its effect on MG132- and Bortezomib-mediated apoptosis. As shown in Figure 2D, 10 μM Z-VAD-FMK had little effect on apoptosis, 20 μM Z-VAD-FMK blocked it by 70 to 90%, and 30 and 40 μM Z-VAD-FMK completely prevented MG132- and Bortezomib-induced apoptosis. We additionally evaluated cell viability using a live-dead cell staining assay, and found that a portion of MG132-treated Cas-FL cells were ethidium homodimer positive indicative of cell death, which was not observed in the presence of Z-VAD-FMK (Figure 2E). This further supports the notion that proteasome inhibition-induced cell death in Cas-expressing cells is due to caspase-mediated apoptosis.

### Caspase signaling underlies MG132-induced apoptosis of MEFs

**Figure 3:**
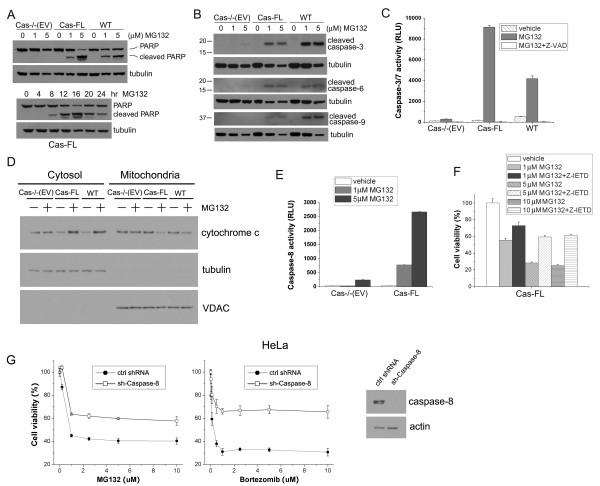
**MG132 induces caspase cascade activation in Cas-expressing cells**. **(A **and **B) **Immunoblots showing cleavage of PARP (A) or caspases (B) in Cas-/- (EV), Cas-FL and WT MEFs treated with 1 or 5 μM MG132 for 16 hours as indicated. In the bottom panel in (A), Cas-FL cells were treated with 1 μM MG132 for times indicated, to assess time-course for PARP cleavage. **(C) **Caspase-3/7 activity assay was carried out using the Caspase-Glo^® ^3/7 Assay kit in MEFs treated with or without 1 μM MG132 for 16 hours. Sample luminescence was measured, and luminescence activity (RLU) was normalized by undertaking parallel MTS assays and evaluating cell number in untreated samples to reflect caspase activity. **(D) **Cas-/- (EV), Cas-FL and WT MEFs treated with 1 μM MG132 were lysed, and separated into cytosolic and mitochondrial fractions, followed by immunoblot to detect the release of cytochrome c from mitochondria to cytosol. Tubulin and VDAC served as cytosolic and mitochondrial markers, respectively to assess purity of the fractions. **(E) **Caspase-8 activity was determined using the Caspase-Glo^® ^8 Assay kit in MG132-treated Cas-/-(EV) and Cas-FL MEFs, and luminescence was examined as in C. **(F) **The caspase-8 inhibitor Z-IETD-FMK (25 μM) was used with various concentrations of MG132 to determine caspase-8 involvement in Cas-FL MEFs death, as measured by the MTS assay. **(G) **HeLa cells stably transfected with Caspase-8 shRNA or control shRNA were treated with the indicated concentrations of the proteasome inhibitors MG132 or Bortezomib for 16 hours, and cell viability was determined by MTS assay. shRNA-mediated reduction of Caspase-8 expression is shown in the immunoblot.

Our data above indicate that the caspases likely mediate MG132-induced MEF cell death, and we, therefore, analyzed caspase activation in MEFs by immunoblot analysis. As shown in Figure 3A, 1 μM MG132 elicited robust PARP cleavage in both Cas-FL and WT MEFs, while it had little effect on Cas-/- (EV) cells (upper panel). Consistently, PARP cleavage was weakly detected in Cas-FL cells 8 hours after MG132 treatment, and the peak response occurred around 16 hours post-treatment (Figure 3A, lower panel). Cleavage of "effector" caspases-3 and -6, and "initiator" caspase-9 was readily detected in both MG132-treated Cas-FL and WT MEFs but not in Cas-/- (EV) cells (Figure 3B). MG132 concentration as high as 5 μM did not induce cleavage of caspases-3, -6, and -9 in Cas-/- (EV) cells. We confirmed that caspases-3/7 were activated by MG132 in Cas-FL and WT MEFs using a luciferase-based luminescence assay, and this activation was completely blocked by Z-VAD-FMK (Figure 3C).

Release of mitochondrial cytochrome c into the cytosol stimulates formation of the apoptosome to activate caspases-3, -6, -7 and -9. To determine whether mitochondrial alteration occurs in MG132-treated MEFs, we analyzed their cytosolic and mitochondrial fractions by immunoblotting. MG132 treatment significantly increased cytosolic cytochrome c levels in both Cas-FL and WT MEFs but not in Cas-/- (EV) cells, suggesting that the release of cytochrome c from mitochondria was involved in MG132-induced, Cas-dependent apoptosis (Figure 3D). Since caspase-8 also mediates apoptosis and functions upstream of "effector" caspases, we undertook a luminescence-based caspase-8 activation assay and found that caspase-8 activation was dramatically induced in MG132-treated Cas-FL cells, but only weakly induced in Cas-/- (EV) cells (Figure 3E). Treatment with the caspase-8-specific inhibitor Z-IETD-FMK significantly protected Cas-FL MEFs from MG132-induced death in MTS assays, suggesting caspase-8 involvement in this process (Figure 3F). Furthermore, when Caspase-8 expression was reduced by shRNA in HeLa cells, these cells became resistant to both MG132 and Bortezomib as shown in the MTS assay in Figure 3G. Taken together, our studies demonstrate activation of the caspase cascade, including activation of the apical caspase-8, upon MG132-treatment in Cas-expressing cells, but not in cells that are deficient of Cas.

### MG132 induces Cas cleavage in apoptotic cells

**Figure 4:**
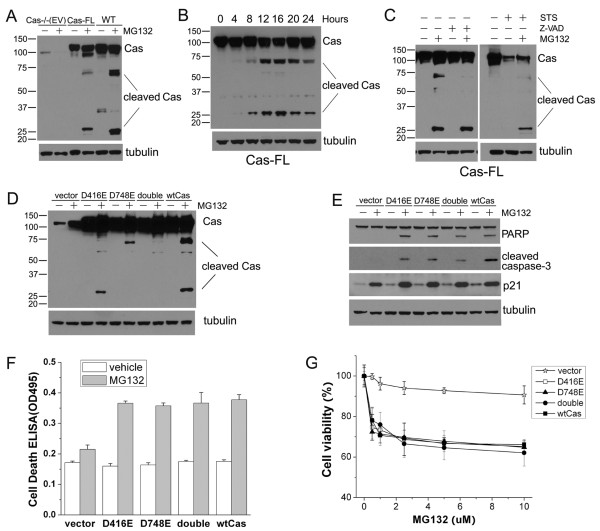
**Cas cleavage during MG132-induced apoptosis**. **(A) **Cell lysates from Cas-/- (EV), Cas-FL and WT MEFs treated with 1 μM MG132 were used in immunoblot analysis to detect Cas cleavage using an anti-p130Cas monoclonal antibody. **(B) **Cas-FL MEFs were treated with 1 μM MG132 for different times, and Cas cleavage was detected by immunoblot as in A. **(C) **Cas-FL MEFs were treated with 1 μM MG132, 25 μM Z-VAD-FMK, or 20 nM staurosporine (STS) alone, or in combination as indicated, and Cas cleavage was detected as in A. **(D **and **E) **Pools of Cas-/- MEFs stably transfected with control vector, or wt Cas, or Cas mutant D416E, or D748E, or the double mutant D416/748E, were treated with 1 μM MG132, and cleavage of Cas (in D) and of PARP and caspase-3 (in E) was detected by immunoblot. Additionally, p21 expression was studied in E by immunoblot. **(F **and **G) **Apoptosis ELISA assay (in F) and MTS assay (in G) were used to examine cell viability of MG132-treated Cas -/- MEFs expressing the constructs indicated.

Previous studies have reported that several focal adhesion proteins, including FAK, paxillin and Cas, undergo cleavage (and subsequent degradation) during cellular apoptosis [13,16]. Here, we found that Cas cleavage takes place in Cas-FL cells and WT MEFs upon MG132 treatment, yielding multiple fragments including the Cas-CT fragment (Figure 4A). Time-course analysis showed that this fragment was detectable 4 hours after MG132 treatment in Cas-FL MEFs, markedly increased thereafter, and reached maximal levels at 16 hours post-treatment (Figure 4B), in concordance with the patterns of PARP cleavage and cell apoptosis (Figures 3A and 2B, respectively). Overall, these observations suggest a positive correlation between Cas cleavage, caspase activation and cellular apoptosis.

Since Z-VAD-FMK prevents MG132-induced cell death (Figures 2A, E and 3C), we next investigated its effect on Cas cleavage. As shown in Figure 4C (left panel), Z-VAD-FMK did not alter the generation nor accumulation of Cas-CT seen in Cas-FL MEFs treated with MG132, suggesting that caspase inhibition alone is not sufficient to prevent Cas-CT generation and accumulation in proteasome-inhibited cells. In control experiments, treatment with staurosporine, a potent inducer of apoptosis, caused severe cell death in Cas-FL cells, resulting in significant degradation of the full-length Cas protein. Interestingly, Cas-CT was hardly detected in these cells, and levels of full-length Cas were greatly reduced (Figure 4C, right panel). In the presence of MG132, however, proteasome-mediated Cas degradation was prevented and subsequently more intact full-length Cas and Cas-CT were detected in staurosporine-treated cells (Figure 4C, right panel). These data combined indicate that both intact Cas and Cas fragments are subject to proteasomal degradation during apoptosis (induced here by either MG132 or staurosporine treatment) and that upon proteasome inhibition, Cas and Cas fragments (including Cas-CT) appear to accumulate in cells.

As noted in the Introduction, Cas cleavage at sites Asp^416 ^and Asp^748 ^precedes the generation of the 31 kD Cas-CT fragment during etoposide-induced apoptosis [9]. To determine the putative functional role of Cas-CT in MG132-induced cell death, we stably expressed wt Cas or Cas mutants containing point mutations in either one or both codons at those two sites (D416E or D748E) in Cas-/- MEFs. We then compared MG132-induced signaling events in these transfected MEFs. In agreement with a previous report [9], we found that the cleavage of the p130Cas-D416E mutant in apoptotic cells still took place, which resulted in increased levels of Cas-CT upon MG132 treatment. Both the D748E mutant and the D416/748E double mutant were resistant to such cleavage, and no Cas-CT generation was detected in cells expressing these mutants following MG132 treatment (Figure 4D). Expression of both single and double Cas mutant proteins, as well as of wt Cas, significantly increased MG132-induced cleavage of PARP and caspase-3 in Cas-/- MEFs, indicating promotion of apoptosis (Figure 4E). Consistently, all the three Cas mutants along with wt Cas effectively mediated MG132-induced apoptosis, based on cell death ELISA (Figure 4F) and cell viability (Figure 4G) assays.

Of note, Kim *et al*. have shown that p21^WAF1/CIP1 ^downregulation is responsible for Cas-CT-mediated apoptosis in etoposide-treated HeLa cells [10]. In our study, expression of full-length Cas or of these cleavage-resistant mutants increased p21^WAF1/CIP1 ^expression in both untreated and MG132-treated MEF cells (Figure 4E), rather, suggesting that p21^WAF1/CIP1 ^downregulation does not underlie MG132-induced apoptosis in this cell type. All these results indicate that, unlike the requirement for Cas-CT generation in etoposide-mediated apoptosis, generation of Cas-CT is not necessary for Cas to mediate MG132-induced cell death.

### Autophagy protects Cas-/- (EV) cells from MG132-induced apoptosis

**Figure 5:**
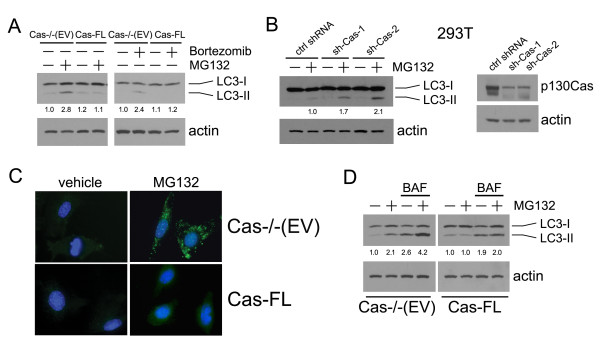
**MG132-induced autophagy in Cas-deficient cells**. **(A) **Cas-/- (EV) and Cas-FL MEFs were treated with 1 μM MG132 or Bortezomib for 16 hours, and LC3-II expression was examined by immunoblot. Relative intensity of LC3-II was calculated by normalizing the untreated Cas-/- (EV) control to a relative intensity of 1.0, and then dividing subsequent LC3-II band intensity by actin band intensity in the same treatment group using densitometry. **(B) **293T cells transiently transfected with Cas shRNAs were treated with 1 μM MG132, and LC3-II expression was examined by immunoblot as in A. **(C) **LC-3 immunostaining in MG132-treated Cas-/- (EV) and Cas-FL MEFs. **(D) **Cas-/- (EV) and Cas-FL MEFs were treated with 1 μM MG132 in the presence or absence of 10 nM Bafilomycin A1 (BAF), and LC3-II expression was examined as in A.

Autophagy can be induced by numerous cellular stresses, such as nutrition deprivation, hypoxia and proteasome inhibition. Conversion of the mammalian protein LC3-I, the homologue of yeast Atg8, to the autophagosome-associated LC3-II is a hallmark of autophagy [17]. When LC3 translocates from the cytosol to the autophagosomal membrane, LC3-II staining goes from diffuse to punctate staining and the protein shows increased gel mobility after lipidation, which is commonly used in autophagy detection [18]. We observed dramatic upregulation of LC3-II following MG132 or Bortezomib treatment in Cas-/- (EV) cells compared to Cas-FL MEFs (Figure 5A), suggesting that proteasome inhibition induces autophagy, but only in cells that lack Cas. To support this notion, shRNA-mediated knockdown of Cas expression in MG132-treated 293T cells also elicited autophagy activation as evidenced by increased LC3-II level in Cas-shRNA expressing cells, but not in control-transfected 293T cells where LC3-II was only weakly detected (Figure 5B). These biochemical observations were further confirmed by immunofluorescence studies, which showed that MG132 treatment induced punctate appearance of endogenous LC3 in Cas-/- (EV) cells, while LC3 remained diffusely distributed in MG132-treated Cas-FL MEFs (Figure 5C).

Diminished levels of the p62 protein represent another widely used marker for autophagy induction [19]. As shown in Additional file 2, we observed that MG132 treatment results in a reproducible and significant decline in p62 levels in MG132-treated Cas -/- (EV) cells compared to Cas-FL cells, and in MG132-treated HeLa cells in which Cas expression has been down-regulated by Cas-specific shRNAs, compared to control-shRNA-transfected cells. Finally, we found that Doxorubicin treatment, similar to proteasome inhibitor treatment, results in a more profound induction of autophagy in Cas -/- (EV) cells compared to Cas-FL cells, as measured by LC3-II and p62 immunoblotting (Additional file 3). Taken together, enhanced cell survival in Cas -/- (EV) cells in the presence of MG132 and Doxorubicin appears to correlate with increased levels of autophagy.

Accumulation of LC3-II could be a consequence of either enhanced autophagic flux, or be due to the inhibition of autophagic degradation of LC3 in MG132-treated Cas-deficient cells. Bafilomycin A1 (BAF), which is a specific inhibitor of vacuolar type H+-ATPase and therefore prevents autophagy at a late stage by inhibiting fusion between autophagosome and lysosome, was utilized to assess the autophagic flux in MG132-treated cells. As shown in Figure 5D, treatment with BAF and MG132 significantly increased LC3-II levels as compared to treatment with either MG132 or BAF alone in Cas-/-(EV) cells, suggesting that autophagic flux was indeed increased in Cas-/-(EV) cells that had been treated with MG132. In comparison, BAF caused similar levels of LC3-II accumulation in the presence and absence of MG132 in Cas-FL cells (Figure 5D).

**Figure 6:**
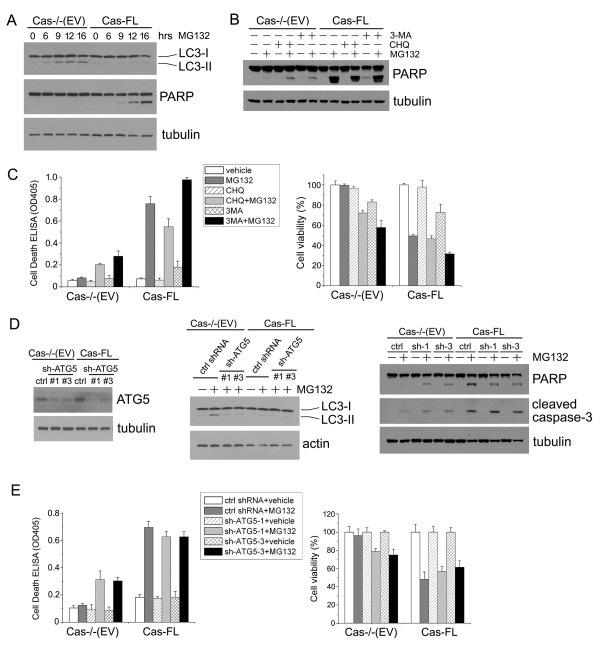
**Autophagy protection of MG132-treated Cas-deficient cells**. **(A) **Time course of MG132-induced autophagy and apoptosis in Cas-/- (EV) and Cas-FL MEFs. Cas-/- (EV) and Cas-FL MEFs were treated with 1 μM MG132 as indicated, and LC3-II expression and PARP cleavage were examined by immunoblot. **(B) **Effect of autophagy on MG132-induced apoptosis. MG132-treated Cas-/- (EV) or Cas-FL cells were assayed by immunoblot to detect the PARP cleavage in the presence of autophagy inhibitors 3-MA (10 mM) or CHQ (20 μM). **(C) **Apoptosis ELISA (left panel) and MTS assay (right panel) were used to examine the effects of autophagy inhibition on MG132-induced cell death in Cas-/- (EV) and Cas-FL MEFs. **(D) **Cas-/-(EV) and Cas-FL MEFs stably transfected with ATG5 shRNAs or control shRNA were treated with 1 μM MG132, and the cleavage of PARP and caspase-3 was examined by immunoblot (right panel). ATG5 expression in the various cell lines as measured by immunoblot is shown in the left panels. The effect of shRNA-mediated knockdown of ATG5 on LC3-II formation is shown in the middle panels, as detected by immunoblot. **(E) **ATG5 shRNA-transfected MEFs were treated with 1 μM MG132, and cell death was examined by the apoptosis ELISA assay (left panel) or by MTS assay (right panel).

It is known that autophagy can protect cells from apoptosis under some circumstances [18]. In a detailed time-course experiment, we found that induction of autophagy took place in Cas-/- (EV) cells as early as six hours post-MG132 treatment, while apoptosis was detected in Cas-FL cells around eight to nine hours after MG132 treatment (Figure 6A). This time-course data suggested that autophagy might play a protective role in MG132-treated Cas-/- (EV) cells and prevent subsequent apoptosis from taking place. Both chemical and genetic approaches were undertaken to study this possibility. Upon treatment of Cas-/- (EV) cells with chemical inhibitors of autophagy, such as chloroquine (CHQ) or 3-methyladenine (3-MA), MG132-induced PARP cleavage was enhanced (Figure 6B), suggesting that autophagy indeed blocks apoptosis in these cells. Inhibition of autophagy had a more limited effect on Cas-FL cell apoptosis, as evidenced by minimal changes in PARP cleavage (Figure 6B). Apoptotic ELISA assay revealed similar results, as inhibition of autophagy by 3-MA or CHQ significantly promoted apoptosis in MG132-treated Cas-/- (EV) MEFs (Figure 6C, left panel). In agreement with these results, the inhibitors significantly enhanced MG132-induced cell death in Cas-/- (EV) MEFs but not in Cas-FL cells, when cell viability assays were utilized as a read-out (Figure 6C, right panel).

To further confirm these results, we used ATG5 shRNAs to block MG132-induced autophagy in Cas-When autophagy was inhibited by these genetic means (Figure 6D, middle panel), increased PARP and caspase-3 cleavage were detected in Cas-/- (EV) MEFs treated with MG132 (Figure 6D, right panel). Consistent with this, blockade of autophagy by ATG5 shRNA significantly promoted Cas-/- (EV) cell death as shown in both apoptotic ELISA assays and in cell viability assays (Figure 6E). Taken together, our results indicate a novel role for Cas in mediating apoptosis downstream of proteasome inhibition, and suggest that Cas may do so, at least partially, by inhibiting MG132-induced autophagy.

## Discussion

Proteasome inhibition promotes cell death via different pathways, depending on cell type and signaling context [20-25]. Proteasome inhibition has also emerged as part of adjuvant therapy to increase sensitivity of cancer cells to chemotherapeutic agents. The docking protein Cas in turn has been previously shown to regulate cell death in response to treatment with various anticancer therapeutics, and thus understanding the potential role that Cas may have in cell death events induced by proteasome inhibition is of great interest.

We show here that Cas expression is required for MG132 to induce cell death via the classical caspase-mediated apoptotic pathway. Previous studies by others have shown that various apoptotic agents stimulate cleavage and subsequent degradation of Cas. Furthermore, it has been suggested that cleavage of Cas might be causal to, rather than consequence of apoptosis [10]. In MG132-induced apoptotic cells, the mechanism of Cas cleavage and the functional significance thereof appear to be more complicated than in the other apoptotic systems. Thus, we show here that MG132 induces Cas cleavage as is seen with other apoptosis inducers. However, MG132 additionally promotes accumulation of intact Cas protein and Cas fragments, including Cas C-terminal fragment (Cas-CT), most likely by increasing their stability. Z-VAD-FMK blocks MG132-induced apoptosis and thus should antagonize Cas cleavage (if caused by caspases), but we nevertheless detect comparable amounts of Cas-CT in Z-VAD-FMK-treated cells, suggesting that proteases other than caspases are involved in Cas cleavage in these conditions (Figure 4C). Thus, we hypothesize that proteasome inhibition plays a dual role in Cas degradation. On one hand, MG132 induces cell apoptosis and activation of caspases and other cellular proteases, promoting Cas cleavage. On the other hand, and perhaps more significantly, MG132 facilitates accumulation of cleaved Cas fragments by increasing their stability. This latter notion is supported by the observation that staurosporine induces robust apoptosis in MEFs, promoting severe Cas degradation with no detectable Cas-CT generation. Simultaneous MG132 treatment, however, significantly increases the accumulation of Cas-CT in staurosporine-treated MEF cells (Figure 4C).

In our studies, Cas cleavage and Cas-CT generation were not found to be required for MG132-induced cell death, as cleavage-resistant Cas mutants D748E and D416/748E effectively promoted MG132-induced cell death without generation and accumulation of Cas-CT (Figure 4D-G). As noted earlier, the Cas-CT cleavage product has been found to induce apoptosis by suppressing p21^WAF1/CIP1 ^expression [10]. Here, we observed significant p21^WAF1/CIP1 ^accumulation in all MEFs treated with MG132, rather, regardless of Cas expression (Figure 4E), further supporting the notion that Cas mediates MG132-induced cell death by a previously unknown mechanism.

As noted in the Background section, overexpression of Cas in cancer cells confers resistance to anticancer drugs such as Doxorubicin. We have tested Doxorubicin in an MTS assay in Cas-/- (EV) and Cas-FL cells, and, curiously, found that similar to proteasome inhibitors, Cas-/- (EV) cells demonstrate noticeable resistance to Doxorubicin-induced cytotoxicity (Additional File 1, Figure S1). We speculate that in cancer cells, where Cas is significantly overexpressed compared to normal physiological levels, Cas overexpression induces aberrant activation of numerous survival pathways, including the PI 3-kinase/Akt cascade, thereby shifting the balance of Bcl-2 family members towards survival [26]. In cells with greatly reduced levels of Cas in turn, we speculate that activation of pathways such as autophagy and possibly others, contributes to drug resistance. As such, different levels of Cas protein appear to contribute to qualitative differences in cellular signaling. Understanding how these qualitative differences in signaling in response to quantitative differences in Cas protein levels take place are of great importance for future studies. Of note, the concept of gene and protein dosage is emerging as a major mechanism to control distinct biological events for some oncogenes [27], and it is intriguing to speculate that this might be true for Cas, too. Clearly, more work is needed to explore this notion.

Autophagy is an adaptive stress response that can both suppress cell death and promote it in the form of autophagic (type II) cell death [28]. Crosstalk between autophagy and apoptosis is complex. In response to the same stress, cells can preferentially undergo either, depending on stimulus intensity and thresholds for either response [18]. When cells are exposed to stress signals, autophagy may protect cells from cell death, and its inhibition can promote cell death. For instance, TRAIL-resistant colon carcinoma cells become sensitized and undergo apoptosis when autophagy is inhibited by specific inhibitors or by Beclin siRNA [29]. In this study, we found that MG132 treatment induces autophagy in Cas-/- (EV) cells and that autophagy appears to protect these cells from apoptosis. This conclusion was supported by the following observations: 1) MG132 treatment of Cas-/- (EV) cells induced conversion of LC3-I to LC3-II, formation of LC3-containing dot structures in the cytoplasm, and reduced levels of p62, all typical markers of autophagy (Figures 5A, C and S2); 2) Increased autophagic flux in these cells was confirmed by treating them simultaneously with Bafilomycin and MG132, which resulted in accumulation of LC3-II in Cas-/- (EV) cells but not in Cas-FL cells (Figure 5D); 3) When autophagy was blocked by two different chemical autophagy inhibitors, namely 3-MA (an inhibitor of the earliest stage of autophagosome formation by inhibition of class III PI3K) or chloroquine ((CHQ), an inhibitor of lysosomal acidification, blocking the fusion between autophagosomes and lysosomes, the final step in the autophagy pathway), or by genetic means using ATG5 shRNA, PARP cleavage was significantly enhanced in Cas-/- (EV) cells treated with MG132 (Figure 6B, D); and 4) In accordance with the result in Figure 5A that autophagy is minimally induced in MG132-treated Cas-FL cells, inhibition of autophagy had limited effects on Cas-FL cell apoptosis, as evidenced by PARP cleavage (Figure 6B, D). At the present time, it remains unclear how autophagy might protect Cas -/- cells from apoptosis. However, we speculate that it might do so by, for example, promoting clearance of toxic protein aggregates formed as a result of proteasome inhibition [18]. Without this clearance mechanism, then, Cas-expressing cells would succumb to apoptotic cell death.

While induction of autophagy appears to protect Cas -/- (EV) cells from proteasome inhibition-induced cell death, it is likely that other mechanisms contribute to the survival, as well. Notably, it has been reported that growth rate influences sensitivity to proteasome inhibitors [30,31]. Indeed, the doubling time for Cas-/-(EV) is 27.6 hours, while it is 20.9 hours for Cas-FL cells during exponential growth phase in tissue culture (Additional file 4). At the present time the underlying mechanisms for this difference or its impact on cell survival remain unclear.

The precise mechanism by which autophagy occurs in Cas-deficient cells but not in Cas-expressing cells in response to proteasome inhibition remains unknown. Cas is an important docking protein downstream of integrin signaling. Integrin ligation to extracellular matrix proteins upon cell attachment promotes cell survival, thus mediating anchorage-dependent cell growth, and Cas has been found to be a positive regulator of these survival pathways. Upon cell detachment and integrin disengagement from the extracellular matrix, cells undergo anoikis (= detachment-induced cell death). Interestingly, cell detachment can also induce autophagy, which offers protection from cell death and limits anoikis [32]. Thus, lack of integrin signaling due to cell detachment, or due to reduced levels of an integrin signaling molecule, such as Cas as shown here, appears to induce autophagy. At the present time, the molecular link between reduced integrin signaling and enhanced autophagy is lacking [33]. The focal adhesion protein FIP200 interacts with ATG1, and becomes distributed to autophagosomes under starvation conditions. In FIP200-deficient cells, autophagy induction by various treatments is abolished, and both stability and phosphorylation of ATG1 are impaired [34]. Similarly, the focal adhesion protein paxillin can be phosphorylated by ATG1 and becomes redistributed from focal adhesions to the cytoplasm along with vinculin in mouse fibroblasts induced to undergo autophagy by nutrient deprivation [35]. Furthermore, paxillin is found to be required for autophagosome formation, but that role is not dependent on integrin signaling [35]. Interestingly, the Cas SH3 domain reportedly targets Cas to focal adhesions by interacting with paxillin (and FAK) [36]. Thus, translocation of the Cas-paxillin complex out of focal adhesions, or release of paxillin from the complex, may be important for autophagy to take place. This notion is supported by our preliminary studies, where we have observed that Cas -/- MEFs reconstituted to express a form of Cas that lacks the SH3-domain (Cas-ΔSH3 MEFs) demonstrate reduced PARP cleavage in response to MG132-treatment compared to Cas-FL MEFs (data not shown). Understanding whether this observation is tied to autophagy, to Cas-paxillin complex formation, and uncovering how Cas regulates autophagy will be an important future objective in our studies.

## Conclusions

Our study demonstrates a previously unidentified, novel role of Cas in mediating cellular sensitivity to proteasome inhibition and, perhaps, to other apoptotic stimuli and sheds light on apoptosis-autophagy crosstalk. Further studies on the mechanisms underlying the role of Cas (and potentially other focal adhesion proteins) in regulating apoptosis and autophagy could provide important insights into molecular switches governing cell death and survival.

## Methods

### Antibodies and reagents

Antibodies against PARP, caspase-3, -6, and -9, cytochrome c, LC3 and ATG5 were from Cell Signaling (Beverly, MA, USA); anti-p130Cas monoclonal antibody used in immunoblot analysis was from Transduction Laboratories (Lexington, KY, USA); anti-tubulin monoclonal antibody was purchased from EMD Biosciences (San Diego, CA, USA); anti- p21^WAF1/CIP1 ^antibody was from Santa Cruz Biotechnology (Santa Cruz, CA, USA); and anti-p62 and anti-actin antibodies were from Sigma (St. Louis, MO, USA). MG132, Bortezomib, Bafilomycin A1, Doxorubicin, Z-VAD-FMK, and Z-IETD-FMK were purchased from EMD Biosciences.

### Cell culture and establishment of stably transfected lines

Cas knock-out MEF cells (Cas-/-), and Cas-/- MEFs transfected with empty vector (Cas-/- (EV)), or reconstituted with wild-type (wt) full-length Cas (Cas-FL) were generously provided by Dr. Sakai and have been described before [37]. Cleavage-resistant Cas mutants were created by replacing aspartic acid residues D416 and D748 in the motifs DVPD^416^G and DSPD^748^G [9] with glutamic acid residues using the Quick Change Mutagenesis Kit (Stratagene, La Jolla, CA, USA). Cas-/- MEFs were cotransfected with empty vector, with wt Cas, or with plasmids encoding the Cas cleavage-resistant mutants, along with pBabe-puro using Fugene HD reagent (Roche, Indianapolis, IN, USA) and selected in puromycin. Transfectant pools were used for experiments. Cas and ATG5 shRNAs were obtained from OriGene (Rockville, MD, USA). 293T cells were transiently transfected with Cas shRNA constructs, and then were used in the assays 24 to 48 hours post transfection. MEFs were transfected with ATG5 shRNA, and then selected in the presence of puromysin to obtain stable lines. All cells were cultured in DMEM containing 10% FCS and 1% glutamine. Cells were routinely treated with 1 μM MG132 in growth medium overnight (16 hours) prior to analysis if not otherwise indicated.

### MTS assay

Cell viability was determined using a CellTiter 96 AQueous Cell Proliferation Assay Kit (Promega, Madison, WI, USA), which is based on measuring metabolic conversion of MTS into aqueous soluble formazan. Briefly, cells grown in 96-well plates were treated as described in the figure legends. A total of 20 μl of MTS reagent was added to each well and incubated for two hours, as recommended by the manufacturer. Absorbance at 490 nm, which is proportional to the number of living cells, was monitored using a plate reader (BioTek, Winooski, VT, USA). Viability of vehicle-treated cells was set at 100%. All the experiments were carried out in triplicates and repeated at least three times. The data are shown as mean ± SE from at least three independent experiments.

### Cell viability assay

MEFs grown on glass bottom microcenter dishes were treated with MG132 and stained with esterase substrate calcein-AM (1:2, 000) and ethidium homodimer-1 (EthD-1, 1:1, 000) in Live/Dead Cell Staining Kit (Invitrogen, Carlsbad, CA), according to the manufacturer's instructions. Images were obtained using a Zeiss fluorescence microscope (Zeiss, Thornwood, NY), and living cells were scored by bright green fluorescence and dead or dying cells by red-orange fluorescence.

### Apoptosis ELISA assay

Apoptosis was quantified using a cell death detection ELISA kit, which measures DNA fragmentation (Roche). MEFs were treated as indicated in the figure legends, and lysed in complete lysis buffer. A total of 5 μg of protein from each sample was used in each apoptosis ELISA, according to the manufacturer's instructions. Absorbance at 405 nm was monitored using a plate reader (BioTek). All the experiments were repeated at least three times, and the data are shown as mean ± SE.

### Caspase activity assay

Activation of caspases 3/7 and caspase 8 was determined by using Caspase-Glo Assay kits (Promega) following the manufacturer's instructions. Cells grown in white-walled 96-well plates were treated with MG132 for 16 hours. A total of 100 μl of Caspase-Glo reagent were added to each well, thoroughly mixed, and incubated for 30 minutes. Sample luminescence was measured with a plate-reading luminometer (Biotek). Luminescence activity reflected by the number of relative light units (RLU) was normalized by undertaking parallel MTS assays and evaluating cell number in untreated samples.

### Immunoblotting

Cell monolayers were treated as described in the figure legends, lysed in modified RIPA lysis buffer (25 mM Tris-HCl, pH7.4, 10% glycerol, 0.2% Triton X-100, 150 mM NaCl, 2 mM EDTA, 2 mM EGTA) containing a protease inhibitor cocktail (Roche), and clarified by centrifugation. Protein concentrations of each sample were determined by Bradford assay. Equal amounts of protein were resolved by SDS-PAGE, transferred to a nitrocellulose membrane, blocked with 3% dry milk (Biorad, Hercules, CA) in TBS-Tween-20 and exposed to specific primary antibodies as described for each experiment. Antibody binding was detected using horseradish peroxidase (HRP)-conjugated goat anti-rabbit or anti-mouse secondary antibodies (Sigma) and enhanced chemi-luminescence (ECL, GE Healthcare, Piscataway, NJ). Tubulin or actin served as the internal loading control.

### Immunostaining of microtubule-associated protein light chain 3 (LC3)

Cells grown in a Lab-Tek^® ^II Chamber Slide System (Nalge Nunc International, Naperville, IL, USA) were treated with or without MG132. After fixation with zinc formalin, cells were permeabilized in 0.2% Triton X-100 for 10 min. Then the cells were blocked in 2% BSA for one hour, followed by the incubation with anti-LC3 antibody at 4°C overnight. Cells were then washed three times with PBS and incubated with Alexa 488-conjugated secondary antibody at room temperature for one hour. After three washings, slides were cover-slipped using FluorSave mounting medium containing DAPI and sealed with nail polish. Images were taken under a Zeiss fluorescence microscope.

### Mitochondrial fractionation

Cytosolic and mitochondrial extracts were prepared by using a mitochondrial isolation kit from Pierce (Rockford, IL, USA) following the manufacturer's instructions for differential centrifugation protocol. In brief, MEFs treated with MG132 overnight were washed once with PBS, and resuspended in 800 μl of buffer A containing protease inhibitor cocktail. After adding buffer B, cells were vortexed and incubated on ice. After adding buffer C and mixing well, the mixtures were centrifuged at 700 g for 10 minutes at 4°C. The supernatants were subjected to another centrifugation at 12, 000 g for 15 minutes at 4°C. The supernatants were then collected and designated as cytosolic fractions. The pellets were washed once with buffer C, then used as mitochondrial fractions. In the following immunoblot, tubulin and VDAC were used as markers for cytosolic and mitochondrial fractions, respectively.

## Abbreviations

3-MA: 3-Methyladenine; BAF: Bafilomycin A1; Cas: focal adhesion protein p130Cas; CHQ: chloroquine; ECL: enhanced chemi-luminescence; EV: empty vector; HEF1: human enhancer of filamentation; HRP: horseradish peroxidase; LC-3: microtubule-associated protein light chain 3; MEF: mouse embryonic fibroblast; MTS: 3-(4: 5-dimethylthiazol-2-yl)-5-(3-carboxymethaloxyphenyl)-2-(4-sulfonyl)-2H-tetrazolium: inner salt; PARP: poly-ADP-ribose polymerase; RLU: relative light units; VDAC: voltage-dependent anion channel; WT: wild type; Z-VAD-FMK: benzyloxycarbonyl-Val-Ala-Asp (OMe) fluoromethyl ketone; Z-IETD: benzyloxycarbonyl-Ile-Glu-Thr-Asp (OME) fluoromethyl ketone.

## Competing interests

The authors declare that they have no competing interests.

## Authors' contributions

MZ and KV together designed the study, interpreted the data and wrote the manuscript. MZ performed the laboratory experiments, while KV supervised the study. Both authors read and approved the final manuscript.

## Supplementary Material

Additional file 1 **Lack of Cas attenuates cell death in response to Doxorubicin**. MEFs grown in 96-well plates were treated with different concentrations of Doxorubicin for 24 hours. Cell viability was measured by MTS assay. The data presented depict the MEAN ± SE from three independent experiments. Click here for file

Additional file 2 **MG132-induced autophagy in Cas-deficient cells as measured by p62 levels**. **(A) **Cas -/- (EV) and Cas-FL cells were treated or not with 1 μM MG132 for 16 hours, and p62 expression was examined by immunoblot. Relative intensity of p62 was calculated by normalizing the untreated control in Cas -/- (EV) cells to a relative intensity of 1.0, and then dividing subsequent p62 band intensity by actin band intensity in the same treatment group using densitometry. (B) HeLa cells transiently transfected with Cas shRNAs or control shRNA were treated with 2.5 μM MG132 for 24 hours, and then assayed for p62 levels. The results (normalized to the untreated control) are presented as in panel A. Click here for file

Additional file 3 **Doxorubicin-induced autophagy in Cas-deficient cells**. Cas -/- (EV) and Cas-FL cells were treated or not with 2 μM Doxorubicin for 24 hours, and the expression of p62 and LC3-II was examined by immunoblot. Relative intensities of p62 and LC3-II were calculated by normalizing the untreated control in Cas -/- (EV) cells to a relative intensity of 1.0, and then dividing subsequent p62 or LC3-II bands intensity by actin bands intensity in the same treatment group using densitometry. Click here for file

Additional file 4 **Growth rate of Cas-/- (EV) and Cas-FL cells**. Cas-/- (EV) and Cas-FL cells were cultured at initial plating density of 2 × 10^4 ^cells/60 mm dish for 0 to 72 h. Cells were harvested at the indicated time points and counted using an automated cell counter. Population doubling times were calculated using Doubling Time Software v1.0.10 http://www.doubling-time.com. Click here for file
