# Supplementary figures and images for: The docking protein p130Cas regulates cell sensitivity to proteasome inhibition
Source: BMC Biol. 2011 Oct 28;9:73. doi: 10.1186/1741-7007-9-73 (PMC3215977; doi:10.1186/1741-7007-9-73)

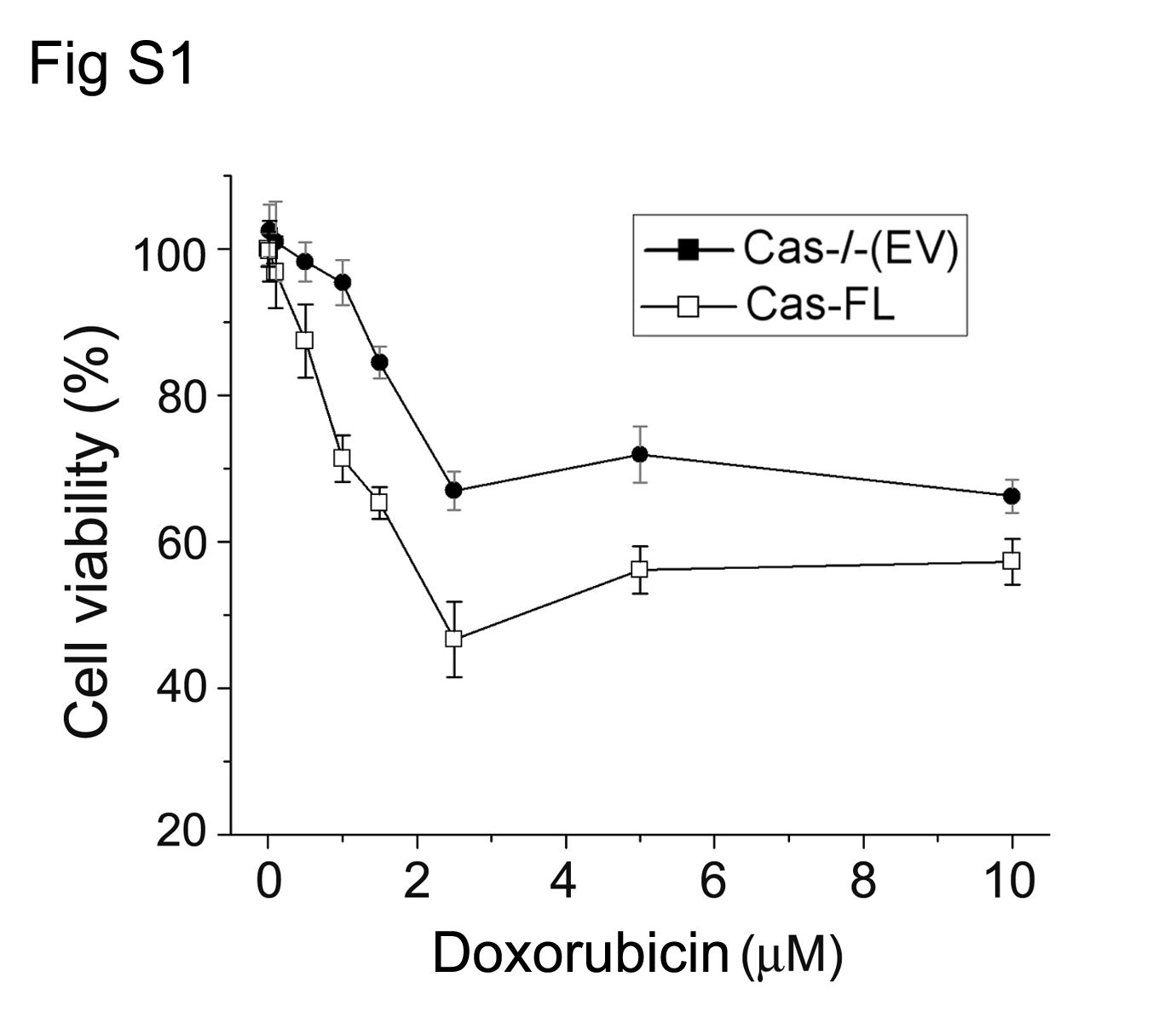

Supplement: Additional file 1 — Lack of Cas attenuates cell death in response to Doxorubicin. MEFs grown in 96-well plates were treated with different concentrations of Doxorubicin for 24 hours. Cell viability was measured by MTS assay. The data presented depict the MEAN ± SE from three independent experiments. [file 1741-7007-9-73-S1.TIFF]

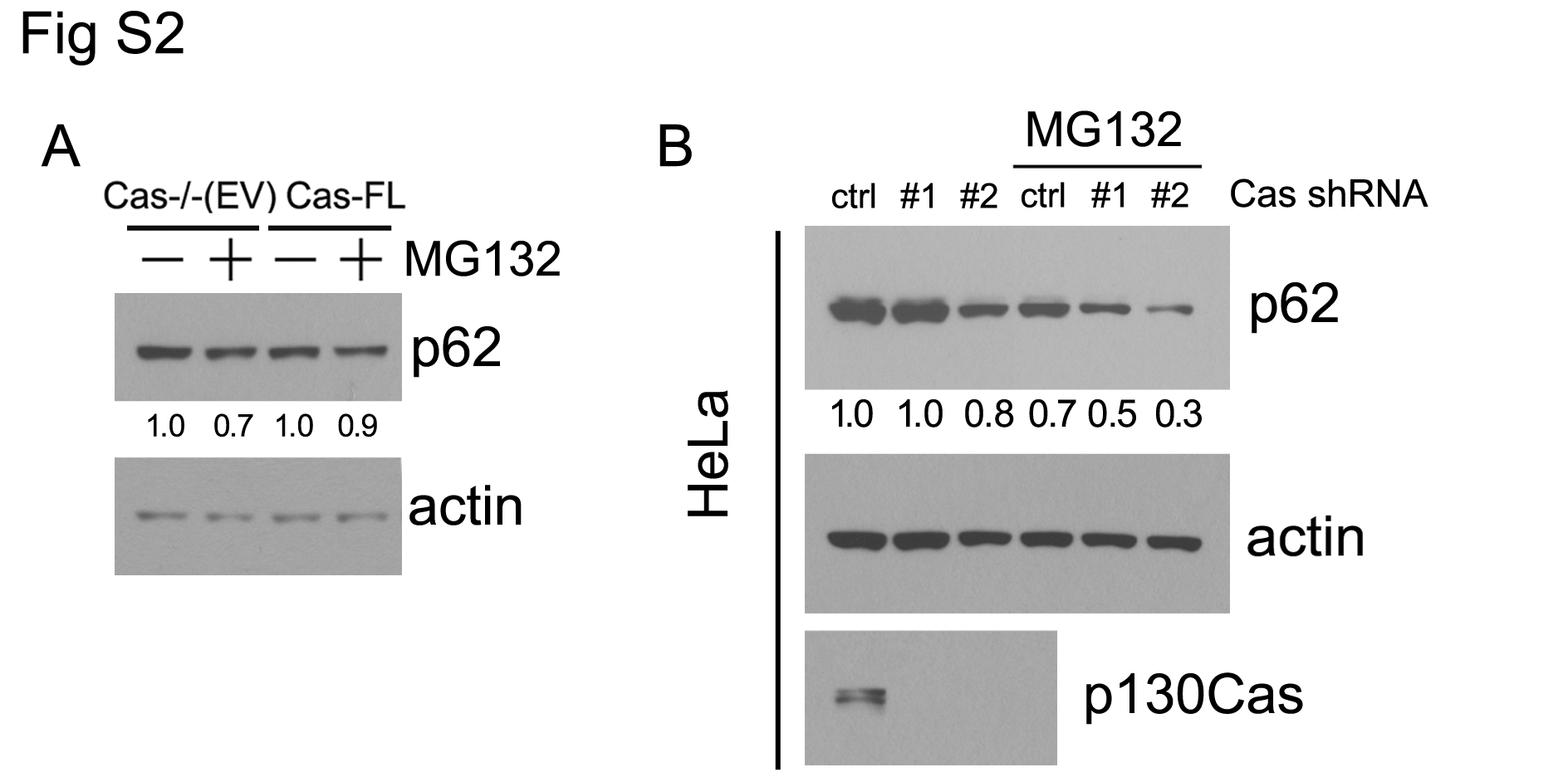

Supplement: Additional file 2 — MG132-induced autophagy in Cas-deficient cells as measured by p62 levels. (A) Cas -/- (EV) and Cas-FL cells were treated or not with 1 μM MG132 for 16 hours, and p62 expression was examined by immunoblot. Relative intensity of p62 was calculated by normalizing the untreated control in Cas -/- (EV) cells to a relative intensity of 1.0, and then dividing subsequent p62 band intensity by actin band intensity in the same treatment group using densitometry. (B) HeLa cells transiently transfected with Cas shRNAs or control shRNA were treated with 2.5 μM MG132 for 24 hours, and then assayed for p62 levels. The results (normalized to the untreated control) are presented as in panel A. [file 1741-7007-9-73-S2.TIFF]

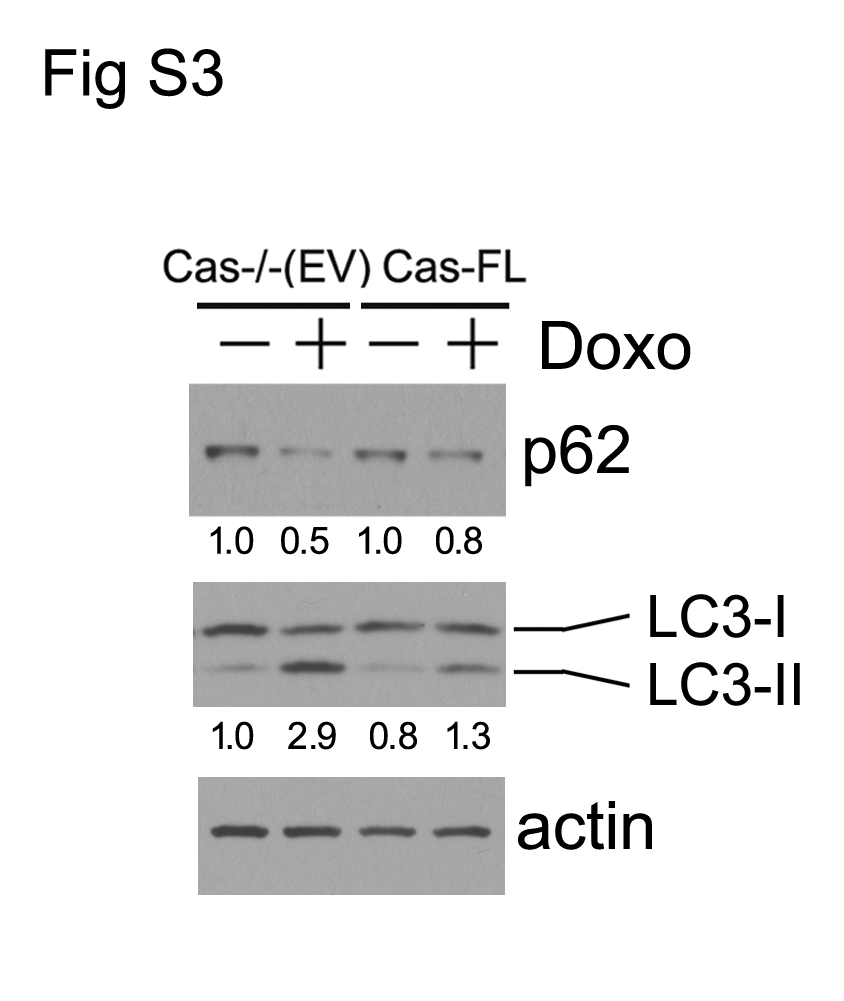

Supplement: Additional file 3 — Doxorubicin-induced autophagy in Cas-deficient cells. Cas -/- (EV) and Cas-FL cells were treated or not with 2 μM Doxorubicin for 24 hours, and the expression of p62 and LC3-II was examined by immunoblot. Relative intensities of p62 and LC3-II were calculated by normalizing the untreated control in Cas -/- (EV) cells to a relative intensity of 1.0, and then dividing subsequent p62 or LC3-II bands intensity by actin bands intensity in the same treatment group using densitometry. [file 1741-7007-9-73-S3.TIFF]

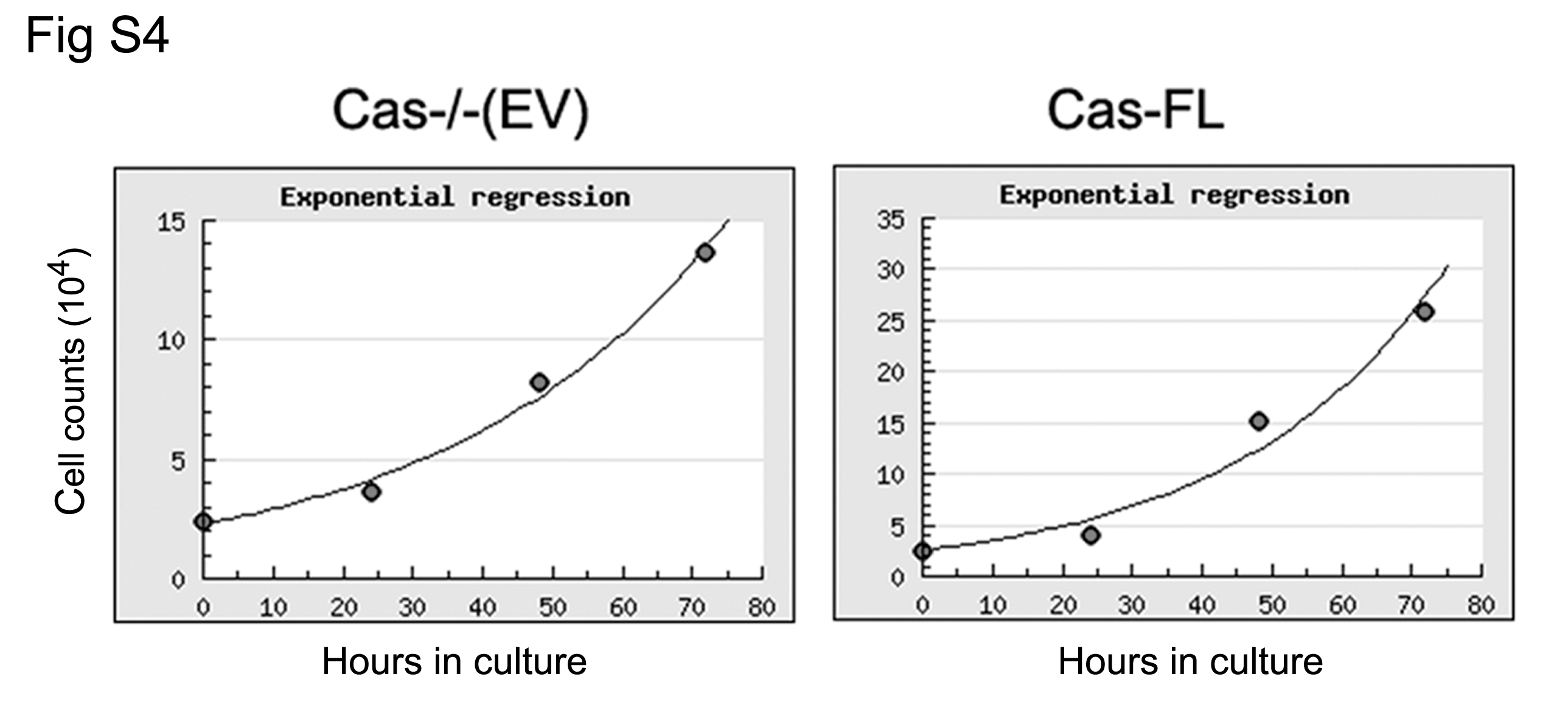

Supplement: Additional file 4 — Growth rate of Cas-/- (EV) and Cas-FL cells. Cas-/- (EV) and Cas-FL cells were cultured at initial plating density of 2 × 10^4^ cells/60 mm dish for 0 to 72 h. Cells were harvested at the indicated time points and counted using an automated cell counter. Population doubling times were calculated using Doubling Time Software v1.0.10 http://www.doubling-time.com. [file 1741-7007-9-73-S4.TIFF]
